# Applications of Green Carbon Dots in Personalized Diagnostics for Precision Medicine

**DOI:** 10.3390/ijms26072846

**Published:** 2025-03-21

**Authors:** Habtamu F. Etefa, Francis B. Dejene

**Affiliations:** Department of Chemical and Physics Science, Walter Sisulu University, Private Bag X-1, Mthatha 5117, South Africa; fdejene@wsu.ac.za

**Keywords:** GCDs, drug delivery systems, theranostics, genomics, proteomics

## Abstract

Green carbon dots (GCDs) have emerged as a revolutionary tool in precision medicine, offering transformative capabilities for personalized diagnostics and therapeutic strategies. Their unique optical and biocompatible properties make them ideal for non-invasive imaging, real-time monitoring, and integration with genomics, proteomics, and bioinformatics, enabling accurate diagnosis and tailored treatments based on patients’ genetic and molecular profiles. This study explores the potential of GCDs in advancing individualized patient care by examining their applications in precision medicine. It evaluates their utility in non-invasive diagnostic imaging, targeted therapy delivery, and the formulation of personalized treatment plans, emphasizing their interaction with advanced genomic, proteomic, and bioinformatics platforms. GCDs demonstrated exceptional versatility in enabling precise diagnostics and delivering targeted therapies. Their integration with cutting-edge technologies showed significant promise in crafting personalized treatment strategies, enhancing their functionality and effectiveness in real-time monitoring and patient-specific applications. The findings underscore the pivotal role of GCDs in reshaping healthcare by advancing precision medicine and improving patient outcomes. The ongoing development and integration of GCDs with emerging technologies promise to further enhance their capabilities, paving the way for more effective, individualized medical care.

## 1. Introduction

Green carbon dots (GCDs), quantum dots (QDs), and graphene derivatives are all nanomaterials with unique properties and applications [1]. Below is a detailed comparison of these materials regarding efficacy, safety, and cost-effectiveness, followed by a table summarizing their competitive advantages and disadvantages. As seen in Table 1, green carbon dots (GCDs), quantum dots (QDs), and graphene derivatives each offer unique properties and applications. Still, they differ significantly in efficacy, safety, cost-effectiveness, and limitations. GCDs are highly biocompatible, tunable in photoluminescence, and eco-friendly, making them ideal for bioimaging, sensing, and catalysis, though they have lower brightness and limited electrical conductivity. In contrast, QDs exhibit superior brightness, narrow emission spectra, and high stability, but their high toxicity, biocompatibility issues, and costly production limit their applications, particularly in biomedical fields. Graphene derivatives excel in electrical conductivity, mechanical strength, and surface area, making them suitable for electronics and energy storage. Still, they face moderate toxicity, environmental concerns, and energy-intensive synthesis challenges. GCDs stand out for their safety, low cost, and scalability. At the same time, QDs and graphene derivatives offer superior performance in specific applications but are hindered by toxicity and higher production costs.

As seen in Table 2, the green synthesis of carbon dots offers significant advantages over conventional methods in terms of energy consumption, environmental impact, and efficiency. Green synthesis operates at lower reaction temperatures (100–200 °C) compared to conventional high-temperature pyrolysis (300–800 °C), resulting in approximately 50–70% lower energy input and 60–75% reduced energy consumption per gram (0.5–1.5 kWh/g vs. 2–5 kWh/g). Additionally, the synthesis duration is significantly shorter (1–4 h vs. 6–12 h), enhancing efficiency. Environmentally, green synthesis utilizes renewable raw materials like biomass and waste, as opposed to non-renewable fossil fuels, and employs non-toxic solvents such as water, contrasting with the toxic organic solvents used in conventional methods. Waste generation is also drastically reduced, with minimal biodegradable by-products compared to significant hazardous waste, achieving an 80–90% reduction. Overall, green synthesis is a more sustainable, energy-efficient, and environmentally friendly approach to producing carbon dots.

Carbon dots are a relatively new class of carbon nanomaterials, first reported in the early 2000s [33]. They are typically defined as quasi-spherical nanoparticles with sizes below 10 nanometers, composed primarily of carbon atoms, with traces of oxygen, hydrogen, and nitrogen. The photoluminescent properties of CDs have attracted considerable interest, as these materials emit light when excited, making them useful for bioimaging and diagnostic applications [34]. Specifically, GCDs have fluorescence emission in the green spectrum, which is particularly beneficial for in vivo imaging due to low tissue autofluorescence in this range, enabling clearer and more accurate imaging results. The field of precision medicine is on the brink of transformative change due to advancements in nanotechnology, with carbon-based nanomaterials playing an increasingly pivotal role [35]. Among these, carbon dots (CDs), particularly green carbon dots (GCDs), have garnered attention for their unique properties and immense potential in medical applications. GCDs, a subset of carbon-based nanomaterials, are distinguished by their photoluminescent characteristics, eco-friendly synthesis, and biocompatibility, all of which make them suitable for diverse applications in medical science, especially in diagnostics and therapeutics [36,37]. The integration of GCDs into precision medicine holds the promise of more targeted, efficient, and safer healthcare solutions tailored to the individual characteristics of each patient.

Precision medicine, often termed personalized medicine, is a medical approach that emphasizes the customization of healthcare based on the genetic, environmental, and lifestyle differences among individuals [38]. Unlike conventional medicine, which often applies a one-size-fits-all strategy, precision medicine aims to provide more tailored interventions, optimizing therapeutic efficacy while minimizing side effects [39]. This approach requires precise and sensitive tools for disease detection, monitoring, and treatment. The versatility of GCDs, from their tunable optical properties to their robust biocompatibility, positions them as valuable assets in achieving these precision-driven objectives. An attractive feature of GCDs lies in their “green” synthesis routes. Unlike conventional CDs, which are often synthesized using toxic precursors and harsh conditions, GCDs can be produced from eco-friendly precursors through simple, cost-effective, and scalable methods [17]. Biomass-derived sources, including fruit peels, plant extracts, and other waste materials, have been widely explored for GCD synthesis [40]. The optical, chemical, and biological properties of GCDs contribute to their suitability in precision medicine. Their tunable fluorescence properties allow researchers to design GCDs with specific emission wavelengths, tailored to desired applications [41]. This customization is particularly relevant for bioimaging, where selecting the appropriate wavelength minimizes background noise, enhances image clarity, and provides higher resolution in cellular imaging. Additionally, GCDs exhibit high photostability compared to conventional organic dyes, which tend to bleach under prolonged exposure to light [42]. This attribute is crucial for real-time monitoring of biological processes in vivo, allowing researchers to track cellular interactions and therapeutic effects over extended periods. The surface functionalization of GCDs further extends their applicability in precision medicine [13,43]. For example, GCDs functionalized with tumor-targeting molecules can selectively accumulate in cancerous tissues, enabling precise delivery of therapeutics to diseased cells while minimizing off-target effects [44]. This characteristic aligns with the goals of precision medicine, which seeks to enhance therapeutic outcomes by delivering the right treatment to the right patient at the right time.

Moreover, GCDs demonstrate excellent biocompatibility, low toxicity, and high cellular uptake efficiency, making them ideal candidates for in vivo applications [45]. Numerous studies have reported minimal adverse effects of GCDs on living cells and tissues, with evidence suggesting that these nanoparticles are either metabolized or safely excreted from the body [46]. This biocompatibility is critical in precision medicine, where the safety profile of therapeutic and diagnostic agents is paramount. One of the most promising applications of GCDs in precision medicine is in disease diagnosis [47]. Fluorescent imaging, particularly in cancer diagnosis, can benefit greatly from the use of GCDs. Due to their strong luminescence and biocompatibility, GCDs can be employed to mark cancerous cells, providing highly specific and sensitive diagnostic results [48]. Additionally, in bacterial and viral infections, GCDs have been explored as fluorescent tags to visualize pathogens and understand the spread of infection within host cells.

Beyond diagnostics, GCDs offer therapeutic advantages as well. In photodynamic therapy (PDT), GCDs can generate reactive oxygen species (ROS) upon exposure to light, leading to the targeted destruction of cancer cells [49]. This therapy minimizes damage to healthy tissue, aligning with precision medicine’s objective to reduce side effects associated with traditional therapies. The application of GCDs in drug delivery is another area of significant interest. By serving as drug carriers, GCDs can enhance the bioavailability and targeted delivery of therapeutic agents, improving treatment outcomes and reducing the risks associated with systemic drug distribution.

## 2. Fundamentals of Green Carbon Dots

An attractive aspect of GCDs lies in their eco-friendly synthesis methods [48]. Unlike other nanomaterials that may require toxic precursors or harsh reaction conditions, GCDs can be synthesized from natural, renewable sources, such as fruits, vegetables, and biomass waste [50]. For example, citric acid, glucose, and even plant extracts have been used as starting materials to produce GCDs in environmentally friendly processes [48]. Such eco-friendly synthesis approaches make GCDs safer for biological applications and align with the broader goals of sustainable development and green chemistry by minimizing waste and avoiding hazardous reagents. In contrast to many traditional fluorescent dyes and quantum dots, GCDs exhibit low toxicity and are often well tolerated by biological systems [51]. This is partly due to their carbon-based composition, which is more compatible with living tissues than metal-based nanoparticles. As a result, GCDs are considered safer for medical applications, particularly in diagnostics and therapy, where long-term exposure and accumulation of nanoparticles can raise toxicity concerns. In summary, these nanomaterials exhibit environmentally friendly synthesis methods, excellent biocompatibility, and chemical stability [52]. These properties and the capacity for surface functionalization make GCDs particularly well suited for applications in biomedical fields where safety, effectiveness, and sustainability are critical considerations. As research continues, GCDs are likely to play an increasingly important role in advancing diagnostic and therapeutic technologies, particularly within the emerging field of precision medicine. In summary, the fundamentals of GCDs include their unique green fluorescence, environmentally friendly synthesis methods, excellent biocompatibility, and chemical stability [52,53]. These properties and the capacity for surface functionalization make GCDs particularly well suited for applications in biomedical fields where safety, effectiveness, and sustainability are critical considerations. As research continues, GCDs are likely to play an increasingly important role in advancing diagnostic and therapeutic technologies, particularly within the emerging field of precision medicine.

### Top-Down and Bottom-Up Approaches for Carbon Dots

**Figure 1:**
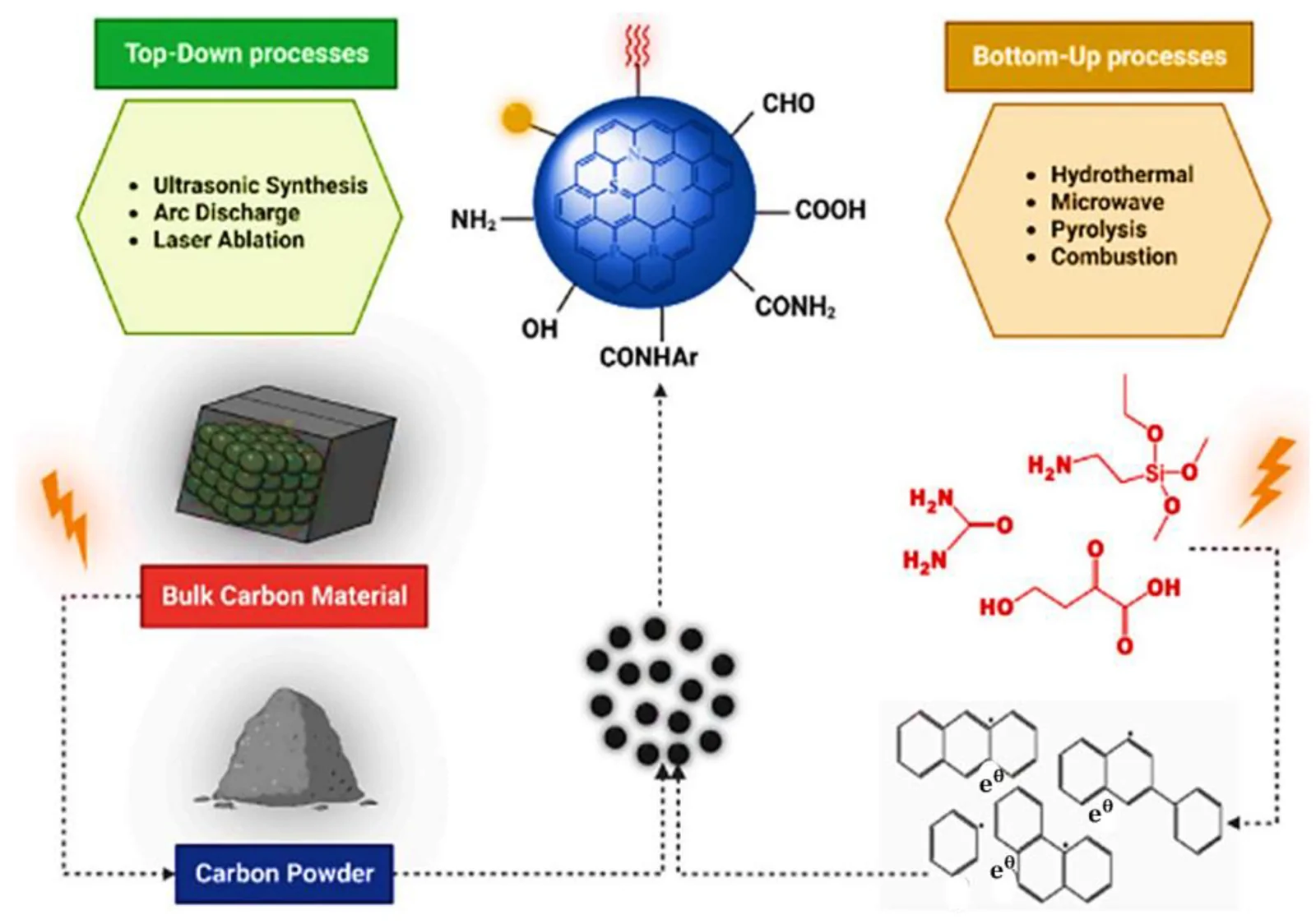
Top-down and bottom-up approaches for the fabrication of carbon dots [57]. Copyright 2023, Elsevier.

GCDs can be synthesized using various methods, primarily divided into two main categories: top-down and bottom-up approaches (seen in Figure 1). Here are the most common methods used to produce GCDs. Carbon dots are produced by top-down synthesis, which breaks up or exfoliates larger carbonaceous materials [54]. The top-down method uses laser ablation, chemical and electrochemical oxidation, arc discharge, and ultrasonication techniques to break down a wide range of carbon nanomaterials (graphite, carbon soot, carbon nanotubes, activated carbon, and nanodiamonds) into smaller carbon nanoparticles. Carbon dots may be produced in a scalable and economical manner using top-down synthesis techniques [55]. They can produce carbon dots in significant amounts and use easily accessible carbon sources. Nevertheless, in contrast to bottom-up approaches, they might have less control over the surface chemistry and functionalization. Bottom-up approaches assemble molecular precursors from smaller carbon units, including carbohydrates, organic acids, polymers, natural products, and biomass, using hydrothermal, carbonization, microwave, solvothermal, pyrolysis, and thermal decomposition procedures, whereas the top-down strategy comprises the breaking down of a wide range of carbon nanomaterials (graphite, carbon soot, carbon nanotubes, activated carbon, and nanodiamonds) into smaller carbon nanoparticles via laser ablation, chemical and electrochemical oxidation, arc discharge, and ultrasonication procedures.

The hydrothermal method is among the most popular synthesis routes for GCDs from the bottom-up approaches due to its simplicity and ability to use eco-friendly precursors. In this method, a carbon source such as citric acid, glucose, or even biomass waste like fruit peels is placed in water or an organic solvent, then heated in a sealed reactor at high temperature and pressure (usually 150–250 °C). The solvothermal method is similar but uses an organic solvent instead of water, which can influence the size and luminescence properties of the resulting GCDs [13]. Microwave-Assisted Synthesis: Another eco-friendly approach is microwave-assisted synthesis, which involves heating carbon sources like citric acid or urea in a microwave [56]. This method is highly efficient, requiring only a few minutes to produce GCDs with consistent properties. The rapid heating causes carbonization, forming nanoparticles with desirable green emission and good dispersibility in aqueous solutions. Ultrasonic Synthesis: In ultrasonic synthesis, high-intensity ultrasound waves are applied to a carbon source, producing localized high temperatures and pressures that break down the precursor into nanoparticles. This method is considered low-energy and relatively straightforward, making it a popular choice for synthesizing GCDs with green emissions. Pyrolysis: Pyrolysis involves the thermal decomposition of carbon precursors in an oxygen-deficient environment. While this method can yield GCDs with excellent photoluminescent properties, it requires precise temperature and atmosphere control to avoid unwanted combustion and obtain the desired size and surface characteristics. Below is a schematic diagram showing various synthesis routes for GCDs, emphasizing the eco-friendly and low-toxicity methods that distinguish these materials in the realm of nanotechnology [57].

## 3. Role of Carbon Dots in Personalized Diagnostics

**Figure 2:**
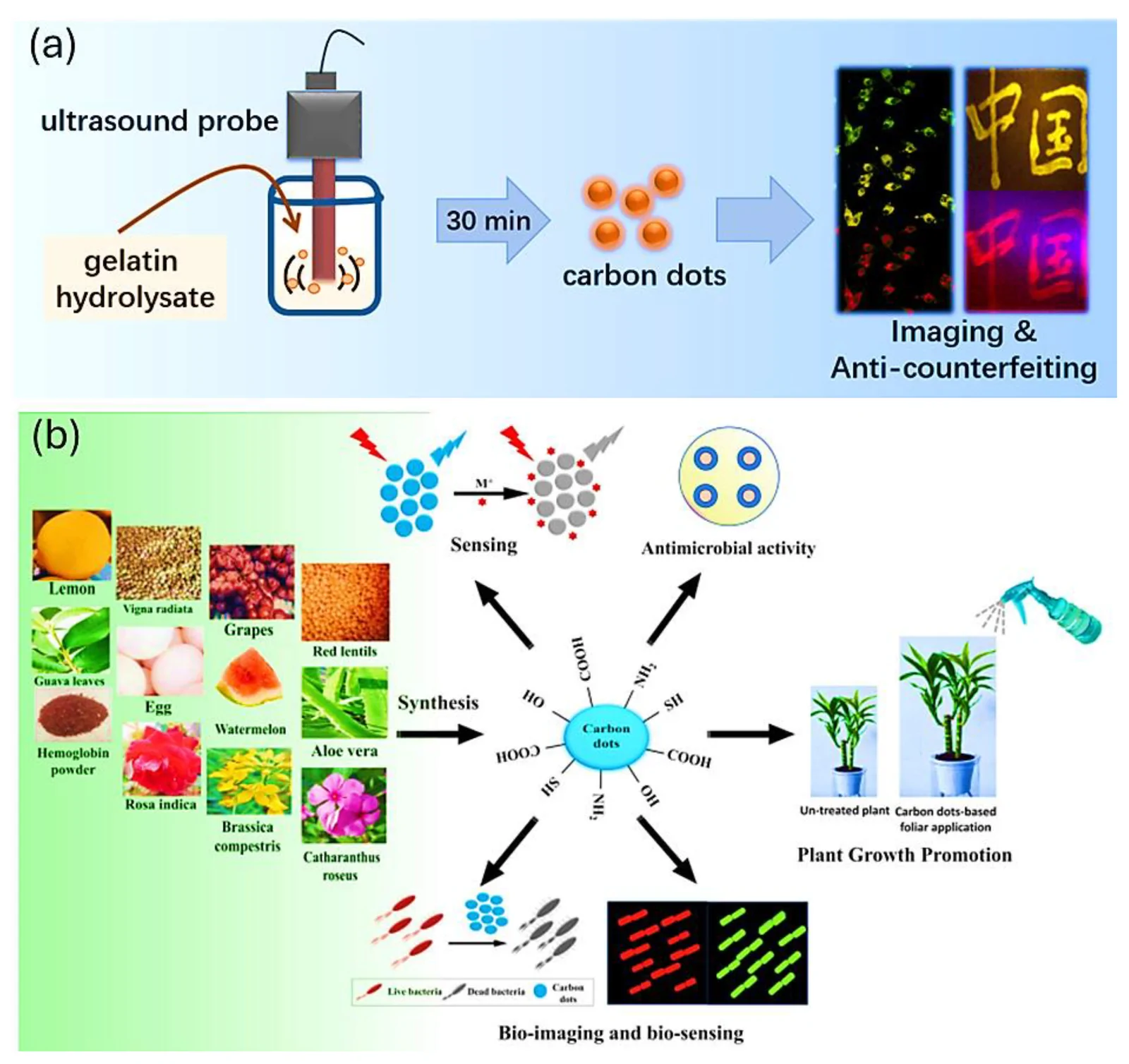
(**a**) Green carbon dots fluorescence imaging and anti-counterfeiting applications [59]. Copyright 2021, Published by American Chemical Society. (**b**) Environmentally friendly sources employed for the Green CDs and their applications for bio-imaging and bio-sensing [60]. Copyright Elsevier (2020).

The field of personalized diagnostics seeks to deliver patient-specific medical insights, improving disease detection, monitoring, and treatment decision-making accuracy [35]. This approach tailors diagnostic tools and therapies based on individual genetic, molecular, and cellular profiles, enabling a more precise understanding of disease at the personal level (Figure 2). Among the novel materials driving advances in personalized diagnostics, GCDs have emerged as promising agents due to their unique properties such as fluorescence, biocompatibility, and eco-friendly synthesis. GCDs’ ability to act as highly sensitive and specific diagnostic probes has positioned them at the forefront of innovations in bioimaging, biosensing, and targeted diagnostics, transforming how diseases are detected and monitored [46,58].

### Photoluminescent Properties in Imaging-Based Diagnostics

One of the defining features of GCDs is their stable green fluorescence, which plays a significant role in diagnostic imaging applications [61]. GCDs, however, exhibit high photostability, maintaining their fluorescence over extended periods. This stability makes them ideal for real-time, high-resolution imaging of cells and tissues, a critical requirement in personalized diagnostics where precise visualization of molecular and cellular details is essential [62]. In cancer diagnostics, for instance, GCDs can be functionalized with tumor-targeting ligands, such as antibodies or peptides that recognize cancer-specific receptors. Once functionalized, these GCDs can selectively bind to cancer cells, providing a high-contrast image of the tumor site while sparing healthy tissue [63]. This selective imaging aligns with the objectives of personalized diagnostics by allowing clinicians to detect even small, early-stage tumors with greater accuracy, leading to better-informed treatment decisions. Additionally, GCDs’ tunable emission properties allow for multicolor imaging, facilitating the simultaneous tracking of multiple biomarkers or cellular processes, which is especially valuable for complex diseases like cancer and autoimmune disorders [64].

## 4. Applications of GCDs in Precision Medicine

**Figure 3:**
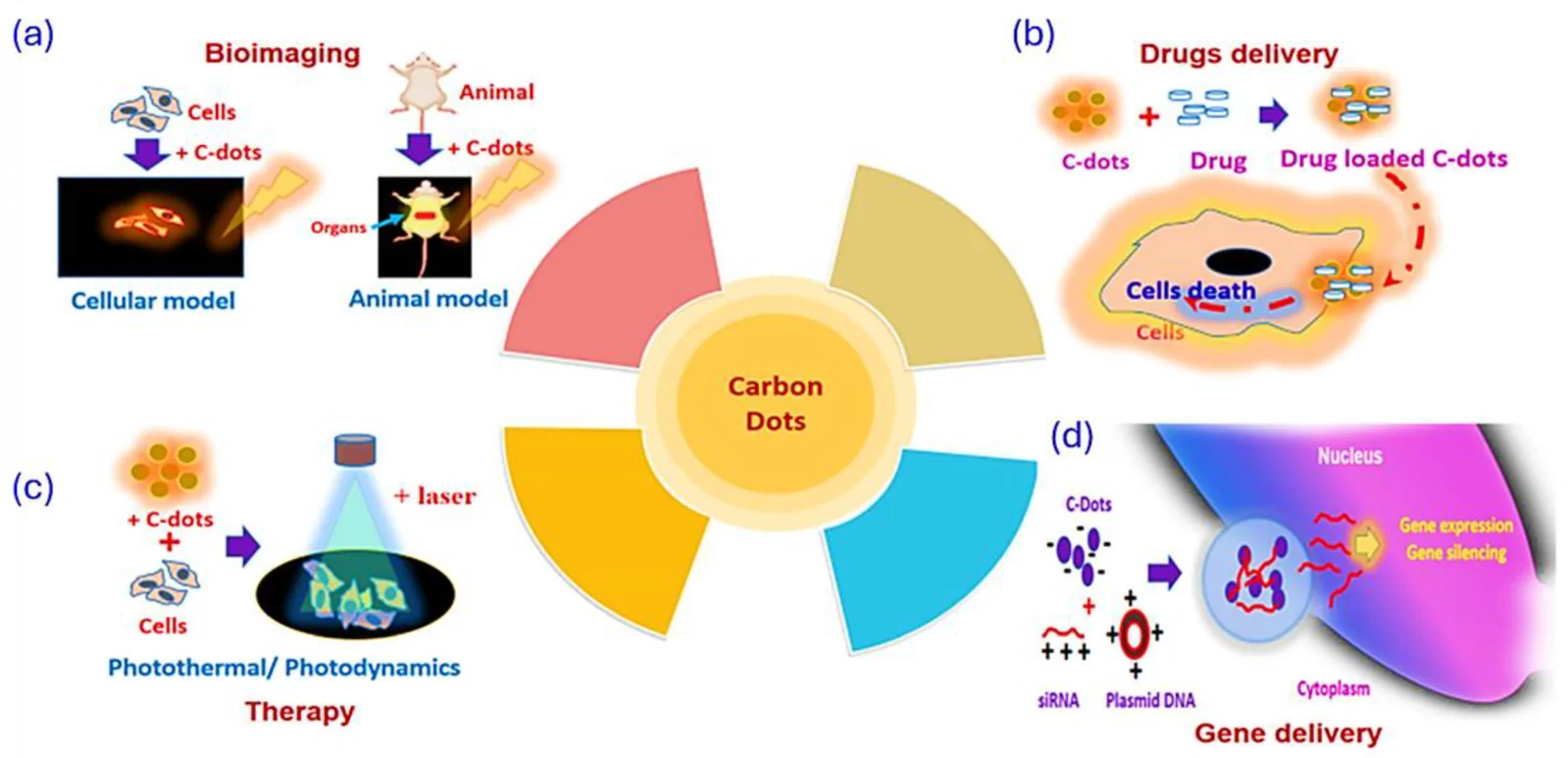
Various biomedical applications of carbon dots: (**a**) bioimaging, (**b**) drug delivery, (**c**) therapy, (**d**) gene delivery [66]. Copyright 2023, mdpi.com.

The benefits and drawbacks of CDs’ use in biomedicine include bioimaging, biosensing, chemical or fluorescence sensing, nanomedicine, electrocatalysis, and photocatalysis. CDs made using different methods have been employed in various applications. These characteristics have made GCDs increasingly valuable in precision medicine, especially in personalized diagnostics, imaging, biosensing, and molecular probing applications. In precision medicine, which seeks to tailor treatments and diagnostics to individual patients based on genetic, molecular, and environmental factors, GCDs are highly adaptable tools that can enhance accuracy, specificity, and sensitivity [65]. GCDs with strong biocompatibility and low cytotoxicity can readily enter cells and disperse throughout the cytoplasmic region, as shown in Figure 3a. CDs can efficiently track and deliver genes or drugs to specific targets, as shown in Figure 3b. Based on Figure 3c, there is a greater need than ever for innovative, improved, focused therapy because of the huge number of deaths brought on by infections and cancer. Photodynamic therapy (PDT) is one potential method to treat various clinical disorders in addition to traditional therapies and drugs. Because CDs can effectively track and deliver genes or medications, as shown in Figure 3d, they can also function as nanocarriers. Branching polyethyleneimine CQDs are one such gene delivery agent. Additionally, CDs are essential for regulating drug release; loading CDs with doxorubicin can restrict drug release in HeLa cells.

### 4.1. Personalized Diagnostics for Cancer

For the next phase of cancer research, diagnostic methods and chemotherapy administration based on nanotechnologies, including carbon dots, may be viable options [67,68]. GCDs have emerged as a promising tool in the field of personalized diagnostics due to their unique properties and eco-friendly synthesis methods [66]. They also exhibit excellent luminescence and biocompatibility, making them ideal for bioimaging applications. Their strong fluorescence allows for high-contrast imaging of biological tissues, aiding in early disease detection [66]. Due to their high stability and tunable fluorescence, GCDs are used in biosensors to detect various biomolecules [14]. This is crucial for personalized diagnostics as it enables the monitoring of specific biomarkers related to individual health conditions. This personalized approach ensures that medications are delivered directly to the affected area, minimizing side effects and improving treatment efficacy [14]. Biomedical applications have advanced significantly thanks to DNA nanostructures with various biological functions. Still missing, nevertheless, is a uniform approach to the effective synthesis of DNA nanostructures [69]. For example, in cancer diagnostics, GCDs functionalized with antibodies against tumor-specific antigens can identify cancer cells in their early stages, allowing clinicians to catch the disease before it progresses. In cardiovascular diseases, GCDs can be modified to detect early biomarkers like troponins or C-reactive proteins, offering a non-invasive means of diagnosis that can lead to faster interventions and improved patient outcomes [70,71]. In personalized diagnostics, the ability to monitor disease progression non-invasively is invaluable. GCDs, due to their stable fluorescence and surface functionalizability, can serve as biosensors that continuously detect disease biomarkers in bodily fluids like blood, urine, or saliva. This enables patients to undergo regular monitoring without invasive procedures, enhancing comfort and compliance with diagnostic protocols [72]. For example, GCD-based sensors can detect circulating tumor DNA (ctDNA) in blood samples [49,73,74,75], providing insights into cancer dynamics and allowing oncologists to adjust treatment plans based on the patient’s real-time condition [76].

#### Imaging for Precision Medicine

**Figure 4:**
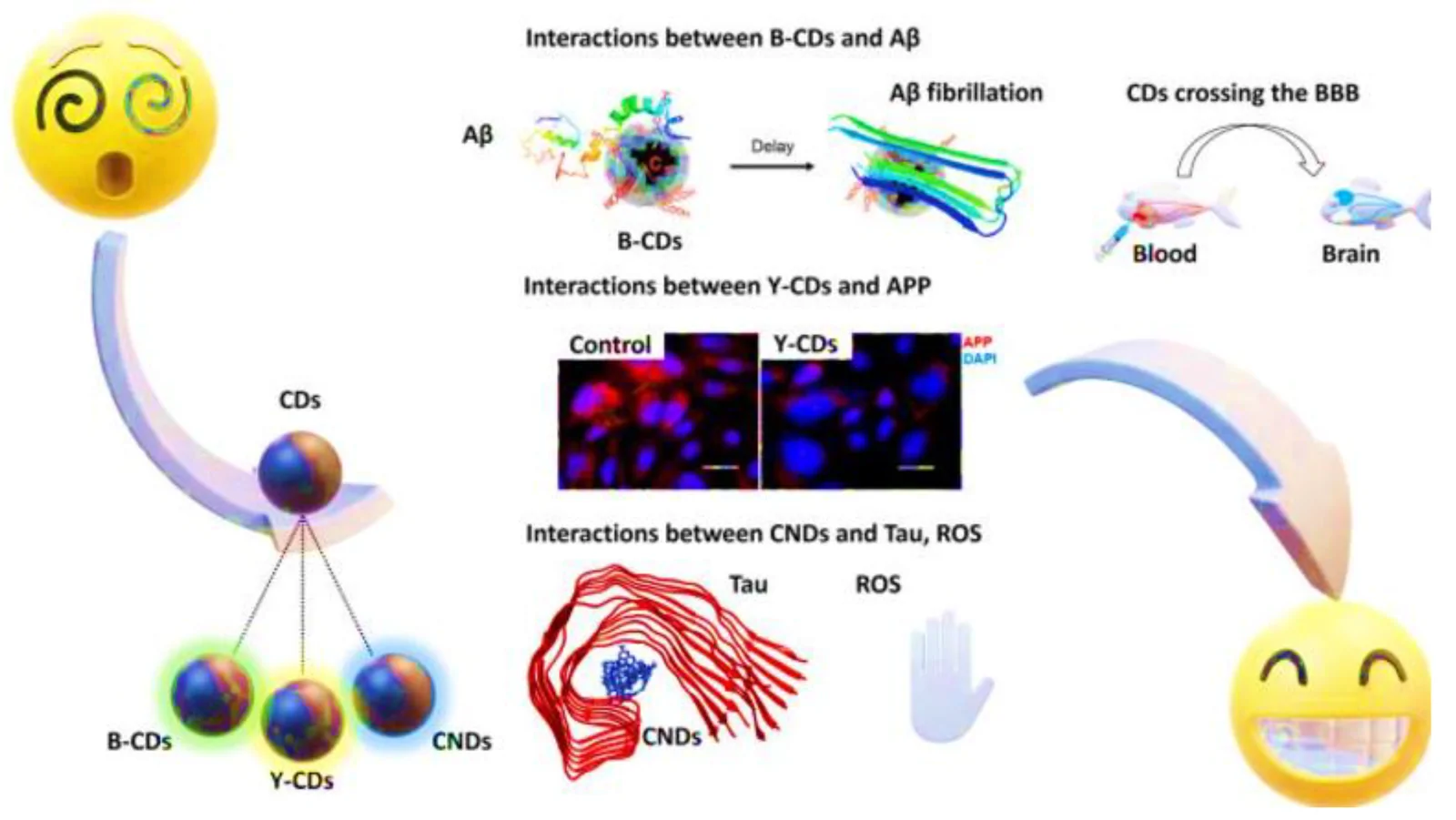
The interactions of various CDs involved in inhibiting different pathogeneses of Alzheimer’s disease (AD) [79]. Copyright 2025, Genetic Engineering and Biotechnology News.

Bioimaging is a critical application area for GCDs in precision medicine. Their stable green fluorescence, biocompatibility, and capacity for functionalization make them highly effective for various imaging modalities [77]. GCDs offer significant advantages over traditional imaging agents, which often suffer from photobleaching or exhibit high toxicity, making GCDs a superior choice for long-term imaging studies. GCDs are particularly effective in cancer imaging, where their high photostability allows for real-time, high-resolution visualization of tumors. This targeted imaging is a marked improvement over conventional imaging agents, which cannot distinguish cancerous cells from healthy tissues accurately [78]. Furthermore, GCDs can be tuned to emit in the green emission range, which minimizes interference from the body’s natural autofluorescence, thereby enhancing imaging clarity and contrast. In addition to emission imaging, GCDs can be engineered for multimodal imaging, integrating fluorescence with other imaging techniques like magnetic resonance imaging (MRI) or computed tomography (CT) [78]. For instance, combining GCDs with magnetic nanoparticles allows for dual fluorescence-MRI imaging, which can provide comprehensive information about the tumor’s location, size, and structural details [46]. This multimodal capability is advantageous in precision diagnostics, as it allows clinicians to gather more information using a single imaging agent, thereby reducing the number of procedures required for an accurate diagnosis and improving patient comfort. Alzheimer’s disease (AD) in Figure 4 is a neurological condition that cannot be cured and becomes worse over time. It gradually erodes a person’s memory and basic cognitive functioning. According to the Alzheimer’s Association, there is presently no cure for Alzheimer’s, making it the sixth most common cause of death in the United States. CDs that shown inhibitory effects on multiple pathogenic aspects of AD, including inflammation, tau, and amyloid, are intriguing nanodrugs in this context. B-CDs suppressed amyloid-beta (Aβ) 42 and 40 fibrillations. Molecular dynamic simulations (Figure 3) demonstrated that B-CDs’ hydrophilic surface enhances their ability to suppress Aβ fibrillation. Additionally, it was discovered that B-CDs successfully prevented beta-secretase 1 activity and postponed the production of Aβ. Following their penetration of cell membranes and entry into cytosolic compartments, Y-CDs markedly reduced the expression and release of Aβ and amyloid precursor protein (APP), respectively (Figure 3). Additionally, CNDs demonstrated a dose-dependent inhibition of microtubule-associated protein tau (MAPT) aggregation. Molecular dynamic simulations revealed a hydrophobic interaction between MAPT and CNDs (Figure 4). CNDs have been shown in a photocatalysis experiment to deactivate reactive oxygen species (ROS) and consume those that are already in the environment, potentially lowering the oxidative stress on the brains of AD patients [79].

### 4.2. Therapy Selection and Monitoring

GCDs are emerging as pivotal tools in precision medicine, particularly in therapy selection and monitoring [46]. This ability to tailor and closely observe therapeutic responses aligns with the core objectives of precision medicine: to optimize treatments based on an individual’s unique biological profile. Among the key applications of GCDs in precision therapy are their roles in drug delivery systems and theranostics. These two applications enable GCDs to function as both therapeutic and diagnostic tools, offering a seamless transition from diagnosis to treatment and facilitating real-time monitoring of therapeutic outcomes [13,65].

#### Drug Delivery Systems

**Figure 5:**
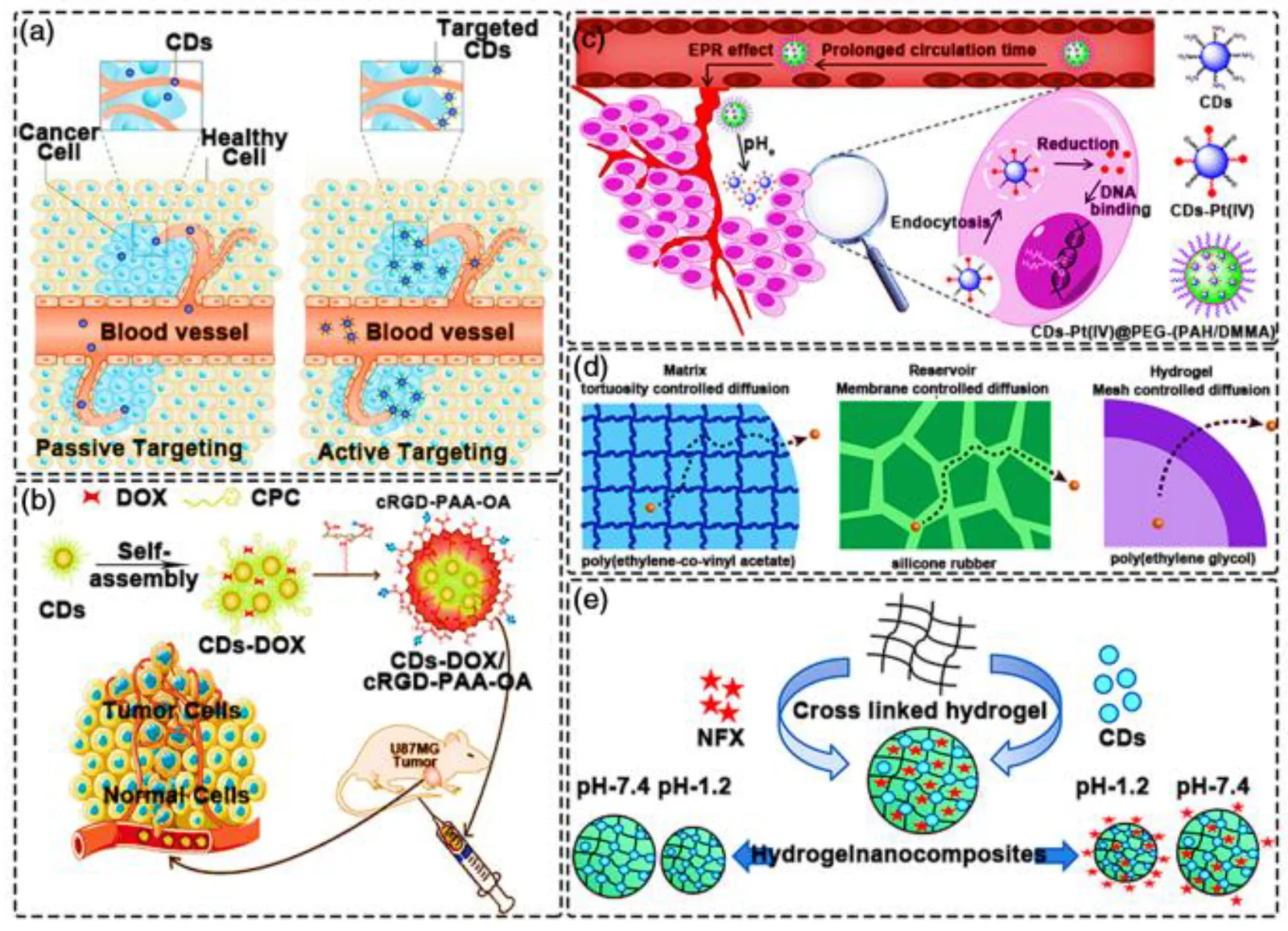
Drug delivery with CDs. (**a**) CDs utilizing the EPR effect to enter a lesion. (**b**) CDs as a delivery platform for DOX. (**c**) CDs being used to deliver cisplatin. (**d**) Mechanisms of controlled drug release. (**e**) CDs-containing hydrogel for the controlled release NFX [96]. Copyright 2022, Wiley Online Library.

In precision medicine, drug delivery systems are vital for ensuring that therapeutic agents reach their intended target while minimizing side effects [80]. GCDs offer significant advantages as drug delivery vehicles, including their small size, tunable surface chemistry, and biocompatibility [46]. As seen in Figure 5a, drugs are incorporated into nanoparticle carriers by encapsulation, adsorption, or binding in nanomaterial-based drug delivery, enabling stable and secure administration within the body [81]. Various types of antibiotic or anticancer medications have been administered successfully thus far [82]. CDs are useful vehicles for the targeted administration of anti-cancer medications (such as doxorubicin (DOX), cisplatin, paclitaxel, docetaxel, camptothecin, daunorubicin, etc.) for cancer treatment because they are simple to modify [83,84]. Because the majority of CDs@drug complexes have cleavable chemical bonds, medicines can be explicitly released in response to various stimuli or the acidic environment of tumor locations [85,86]. CDs can help cancer cells or other diseased cells overcome their drug resistance and improve delivery and controlled release at the target spot.

As seen in Figure 5b, one frequent first-line treatment for many malignancies is DOX, which can be coupled with DNA-related enzymes of cancer cells to intercalate DNA base pairs to prevent their synthesis and replication [87]. The targeted release of DOX in the tumor microenvironment via biocompatible CDs has garnered much interest in recent years. Most of these groundbreaking studies have concentrated on improving the EPR (Enhanced Penetration and Retention) impact to boost medication loading capacity and increase tumor accumulation. Composites of CDs and DOX that exhibit notable tumor targeting, improved anti-tumor activity, and fewer side responses have been created by building CDs with a hydrophilic shell and a hydrophobic carbon core. Because normal CDs have trouble combining with DOX, CDs must be modified on the surface. The changeability of CDs@DOX systems must be carefully considered due to the acidic nature of the tumor site microenvironment [88]. Human glioblastoma cell line (U87MG) tumors were selectively targeted by CDs@DOX by surface charge modification using an octylamine-modified polyacrylic acid (cRGD-PAA-OA) and cRGD coating. Conversely, CDs-based composite materials demonstrated an excellent release profile of DOX over time in in vitro drug release studies (Figure 5b) [89]. Cisplatin (Pt(IV)), in addition to DOX (Figure 5c), is frequently used to treat a variety of cancers [90]. However, the effectiveness of cisplatin-based cancer treatments is typically hampered by acquired and/or intrinsic drug resistance, as well as the severe adverse effects. As a result, creating drug targeting systems with high site specificity and managing drug release rate is crucial. CDs make an excellent delivery system for these medications because of their compact size and simplicity of use. It is important to carefully evaluate using CDs in combination with Pt(IV)-based medications [91]. CDs can fix Pt(IV)-based medications when their surfaces are functionalized appropriately. PEGylation is a popular surface functionalization technique. Via an electrostatic repulsion mechanism, the cisplatin-based CDs platform (CDs-Pt (IV)@PEG-(PAH/DMMA)) can encourage the release of positively charged CDs-Pt (IV) seen in Figure 5c [92].

Controlled and gradual release of pharmaceuticals are still problems with CD-based systems that need to be resolved (Figure 5d), in addition to the quantity of drugs-loaded. Drugs that are rapidly removed or broken down when taken alone should be able to be delivered by an optimal sustained-release mechanism [93]. Additionally, the therapeutic agent must be released consistently during treatment to prevent the drug from being damaged during the release time. According to the previously described reports, medications can be placed onto CDs using various techniques, and they can subsequently be stimulated or released from the CDs in a regulated way [94]. Therapeutic agent typically used in CD-based controlled release systems, polymer matrices, hydrogel networks, or mesoporous silica work to enhance local therapeutic benefits and reduce adverse effects. In Figure 5e, reservoir-controlled release systems limit the release of trapped therapeutic agents through a membrane that controls the rate at which the therapeutic agent diffuses out of the reservoir; (3) The mesh size of the swelling polymer network controls the rate of drug release from hydrogel-based release systems; and (4) Therapeutic molecules diffuse through a tortuous network of interconnected pores created during the phase separation of the drug/excipient and the polymer. As an illustration of the latter, a pH-responsive biodegradable hydrogel-nanocomposite was created using CDs as the cross-linker and an agarose-poly(vinyl alcohol) copolymer. Norfloxacin (NFX), an antibiotic medication, was released from the hydrogel-nanocomposite using zero-order kinetics [95].

Furthermore, GCDs have demonstrated the ability to bypass efflux pumps on cancer cell membranes, often responsible for drug resistance in tumors. By penetrating cancer cells more effectively, GCD-based drug delivery systems can overcome resistance mechanisms and improve the intracellular accumulation of therapeutic agents, thus enhancing treatment success rates. These unique properties make GCDs invaluable in precision medicine, where the ability to precisely target and deliver drugs to specific locations within the body can significantly influence patient outcomes.

## 5. Challenges in the Application of GCDs

### 5.1. Standardization and Scalability of Synthesis

A critical challenge in applying GCDs in clinical settings is the lack of standardized synthesis protocols. While numerous methods for synthesizing GCDs exist, each may yield GCDs with slightly different physicochemical properties, such as size, surface charge, and fluorescence [97]. These variations affect the performance of GCDs, including their bio-distribution, toxicity, and efficacy in targeting specific molecules [98]. Furthermore, the scalability of GCD synthesis remains a concern; ensuring consistent quality, stability, and bioactivity at large production scales is essential for regulatory approval and safe clinical application.

### 5.2. Understanding and Minimizing Toxicity

Although GCDs are generally considered biocompatible and have lower toxicity than traditional carbon-based nanomaterials, their long-term safety remains unclear [99]. The impacts of chronic exposure, accumulation in different organs, and potential for cellular or genetic toxicity are areas that need thorough investigation. Additionally, GCD surface modification, often necessary for targeted delivery or improved stability, may introduce new toxicity risks or unforeseen biological interactions [77]. A comprehensive understanding of GCD toxicity across various biological systems, alongside targeted studies on clearance mechanisms, is essential for addressing regulatory and safety concerns.

### 5.3. Limited Targeting Precision and Delivery Efficiency

While GCDs have shown promise as carriers for drug delivery and gene therapy, achieving high targeting specificity and efficient delivery remains challenging. Often, GCDs struggle with avoiding off-target effects, as their small size and functionalization properties may lead them to accumulate in non-target tissues [100]. GCDs need advanced surface modifications or loading techniques to improve their precision to ensure selective binding to disease-specific receptors. Increasing targeting accuracy enhances therapeutic efficacy and minimizes side effects, which is critical for applications in sensitive areas such as oncology or neurodegenerative disorders [101].

## 6. Future Directions for GCDs in Precision Medicine

### 6.1. Developing Targeted and Functionalized GCDs

Future research on GCDs will likely focus on developing more targeted and functionalized GCDs for improved disease detection and treatment precision [44]. Researchers can enhance GCD selectivity by modifying GCDs with ligands, antibodies, or aptamers specific to disease biomarkers, enabling more accurate localization to disease sites and more effective therapeutic delivery. Additionally, developing stimuli-responsive GCDs that release therapeutic payloads in response to environmental changes, like pH or temperature, could significantly improve the control of drug release, optimizing treatment efficacy and minimizing side effects [102].

#### 6.1.1. Enhancing Biocompatibility and Safety Profiles

As the long-term effects of GCDs in the body are still under research, future studies should prioritize biocompatibility and safety [77]. One approach is to design biodegradable GCDs that break down into non-toxic byproducts after completing their function. Further research into GCD surface chemistry will also be essential, aiming to minimize immune response, improve clearance from the body, and ensure that GCDs remain safe for repeated or prolonged use. This research will be instrumental in moving GCD-based technologies closer to clinical trials and eventual regulatory approval [46].

#### 6.1.2. Advancing Bioinformatics Integration and Real-Time Monitoring Systems

**Figure 6:**
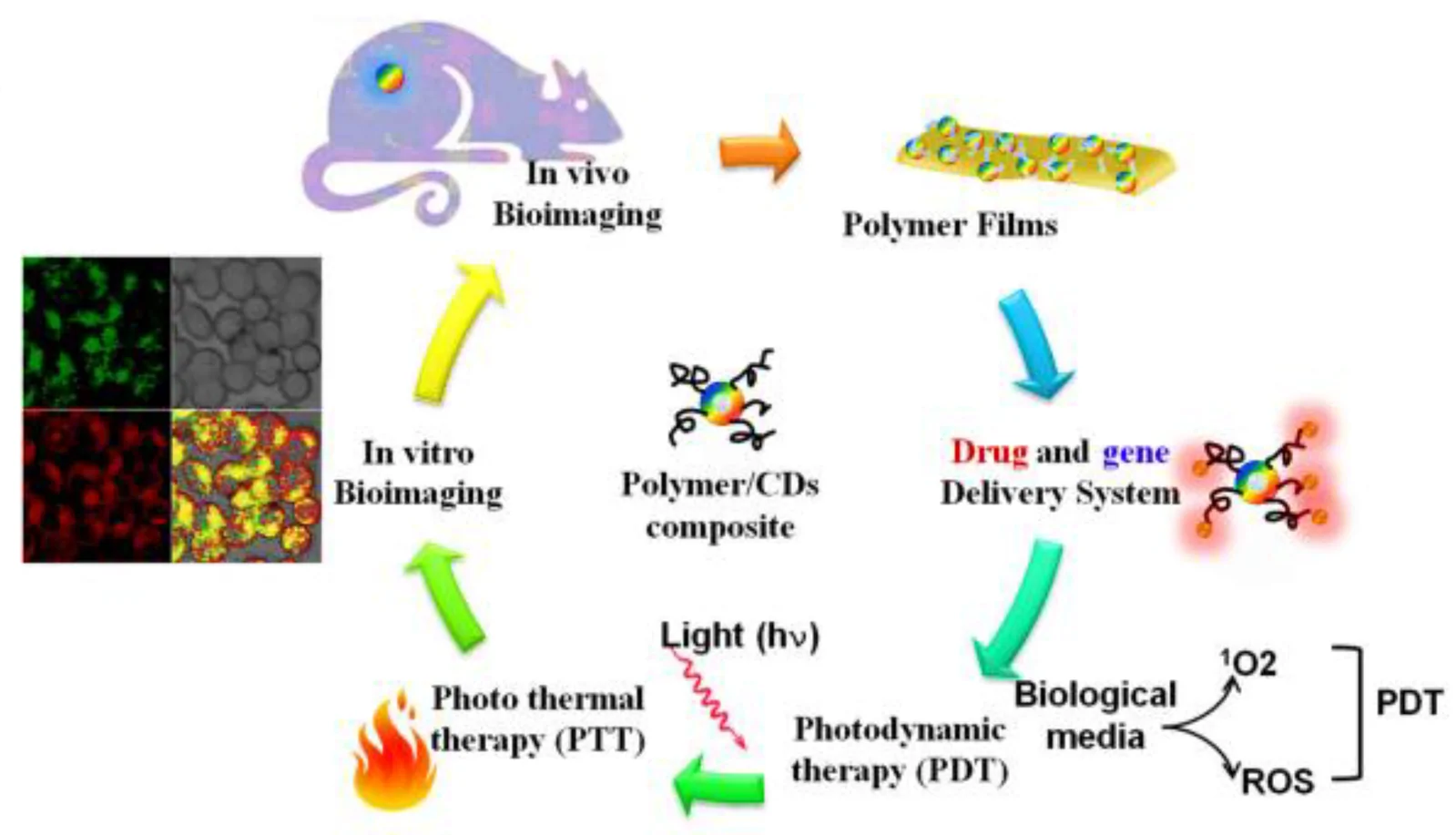
Proposed biomedical application of CD/polymer composite materials [109]. Copyright 2022, mdpi.com.

With further advancements in bioinformatics, GCD-based platforms could enable real-time monitoring and adaptive treatment responses [103]. For instance, integrating GCD-generated data with machine learning models could provide predictive insights on treatment efficacy or disease progression, allowing for personalized adjustments [104]. Developing secure, interoperable data systems will ensure that GCD data can be effectively utilized alongside genomic, proteomic, and clinical data to create a comprehensive patient profile, enhancing the precision of diagnoses and therapies. Finally, GCDs represent a promising frontier in precision medicine, with applications ranging from drug delivery and diagnostics to real-time patient monitoring. However, challenges in standardization, biocompatibility, targeting precision, and bioinformatics integration must be addressed before these materials can be widely adopted in clinical practice. By focusing future research on functionalization, biocompatibility, multi-modal applications, and data integration, the medical community can move closer to realizing the potential of GCDs in delivering highly personalized, efficient, and safe patient care [105]. As seen in Figure 6, the cross-linked polymeric characteristics and easy manufacturing of CDs have stimulated the development of prospective biomedical research applications (e.g., drug delivery system, photodynamic treatment, bioimaging, etc.). Bioinformatics leverages computational tools and algorithms to analyze biological data, transforming raw data into actionable insights [106]. In precision medicine, bioinformatics helps make sense of data from a range of omics fields, including genomics, proteomics, and metabolomics, creating comprehensive patient profiles [107]. By integrating these datasets, bioinformatics enables clinicians to predict disease risk, personalize treatments, and monitor patient responses, all based on a thorough understanding of a patient’s unique biological makeup. In this context, bioinformatics is essential for maximizing the utility of GCDs by interpreting the data they generate in real time [108].

## 7. Conclusions

In conclusion, Green GCDs are a significant advancement in nanotechnology, particularly for personalized diagnostics and precision medicine. Their unique properties, such as small size, biocompatibility, and tunable fluorescence, make them ideal for various biomedical applications. GCDs are synthesized through eco-friendly methods and can be functionalized to target specific biomolecules, enhancing their versatility. In personalized diagnostics, GCDs excel in imaging and biosensing, providing high-resolution insights into disease states and enabling early and precise detection through molecular probing. For therapy selection and monitoring, GCDs offer innovative solutions like drug delivery systems and theranostics, which combine diagnostic and therapeutic functionalities for real-time treatment monitoring. Integrating GCDs with genomics, proteomics, and bioinformatics is advancing personalized treatment strategies by enabling comprehensive molecular profiling and optimizing patient-specific interventions through data analytics and machine learning. Despite their potential, GCDs face challenges such as the need for standardized synthesis methods, long-term biocompatibility assessments, and efficient targeting capabilities. Addressing these challenges through future research is crucial to fully realize the potential of GCDs in clinical settings. Overall, GCDs hold great promise for revolutionizing personalized diagnostics and precision medicine, offering targeted, effective, and responsive healthcare solutions tailored to individual patient profiles. By overcoming current limitations, GCDs could play a pivotal role in advancing precision medicine and improving patient outcomes.

## Figures and Tables

**Table 1:** Competitive Advantages and Disadvantages.

Property	Green Carbon Dots (GCDs)	Quantum Dots (QDs)	Graphene Derivatives
Efficacy	Tunable photoluminescence, High biocompatibility, Easy functionalization [2,3]	Superior brightness, Narrow emission spectra, High stability [4,5]	Excellent electrical conductivity, High mechanical strength, Large surface area [6]
Safety	Low toxicity, Biodegradable, Eco-friendly synthesis [7]	High toxicity (heavy metal-based), Biocompatibility issues [8]	Moderate toxicity, Environmental concerns [9]
Cost-Effectiveness	Low-cost synthesis and scalable production [10]	High production costs, Limited scalability [11]	Moderate cost, Energy-intensive synthesis [12]
Applications	- Bioimaging - Sensing - Catalysis [13,14]	- Displays - Solar cells - Biomedical imaging (limited) [15]	Electronics, Energy storage, and Sensors [16]
Limitations	- Lower brightness compared to QDs - Limited electrical conductivity [17]	- Toxicity concerns - High cost [18]	Toxicity in biological systems, High energy consumption [19]

**Table 2:** The eco-friendly synthesis of green carbon dots with conventional energy consumption and environmental impact methods.

Parameter	Green Synthesis of Carbon Dots	Conventional Methods	Comparison	Refs.
Energy Consumption				
Reaction Temperature	100–200 °C (low-temperature processes)	300–800 °C (high-temperature pyrolysis)	~50–70% lower energy input	[20,21]
Energy Input (per gram)	0.5–1.5 kWh/g	2–5 kWh/g	~60–75% lower energy consumption	[22,23]
Duration of Synthesis	1–4 h	6–12 h	~50–70% shorter synthesis time	[24,25,26]
Environmental Impact				
Raw Materials	Renewable (e.g., biomass, waste)	Non-renewable (e.g., fossil fuels)	100% renewable vs. non-renewable	[27,28]
Solvent Use	Water or mild solvents	Toxic organic solvents	Non-toxic vs. toxic solvents	[29,30]
Waste Generation	Minimal (biodegradable by-products)	Significant (hazardous waste)	~80–90% less waste	[31,32]

## Data Availability

Not applicable.
