# Correction: Etefa, H.F.; Dejene, F.B. Applications of Green Carbon Dots in Personalized Diagnostics for Precision Medicine. *Int. J. Mol. Sci.* 2025, *26*, 2846

**DOI:** 10.3390/ijms27052349

**Published:** 2026-03-03

**Authors:** Habtamu F. Etefa, Francis B. Dejene

**Affiliations:** Department of Chemical and Physics Science, Walter Sisulu University, Private Bag X-1, Mthatha 5117, South Africa; fdejene@wsu.ac.za

## References

In the original publication [1], citation reference 35 was retracted. The author has reviewed and decided that this reference should be removed. With this correction, the order of some references has been adjusted accordingly. The authors state that the scientific conclusions are unaffected. This correction was approved by the Academic Editor. The original publication has also been updated.
